# Clinical application of targeted α-emitter therapy in gastroenteropancreatic neuroendocrine neoplasms

**DOI:** 10.1007/s00535-025-02241-z

**Published:** 2025-04-12

**Authors:** Naoyuki Yamaguchi, Jing-Jing Wei, Hajime Isomoto

**Affiliations:** 1 Department of Endoscopy, Nagasaki University Hospital, 1-7-1 Sakamoto, Nagasaki, Nagasaki 852-8501 Japan; 2 Department of Endoscopy, the First Affiliated Hospital of Fujian Medical University, Cha Zhong Road No.20, Tai Jiang District, Fuzhou, 350004 Fujian China; 3 Department of Endoscopy, National Regional Medical Center, Binhai Campus of the First Affiliated Hospital of Fujian Medical University, Fuzhou, 350004 Fujian China; 4 Division of Gastroenterology and Nephrology, Department of Multidisciplinary Internal Medicine, School of Medicine, Faculty of Medicine, Tottori University, Yonago, 683-8504 Japan

**Keywords:** α-Emitters, Gastroenteropancreatic neuroendocrine neoplasm, Peptide receptor radionuclide therapy

## Abstract

Effective therapeutic strategies for advanced gastroenteropancreatic neuroendocrine neoplasms (GEP-NENs) remain challenging, including a lack of response to therapy and post-treatment relapse. The rapid development of targeted radionuclide therapy (TRT) offers promising data for patients with somatostatin receptor (SSTR)-expressing tumors. This approach exhibits more advantages than somatostatin analog (SSA) therapy, which is primarily effective for well-differentiated and slow-growing GEP-NENs. Fortunately, some clinical studies on peptide receptor radionuclide therapy (PRRT) labeled with α-emitting radionuclides for GEP-NENs patients showed effective results for those with more advanced GEP-NENs, or those with malignant metastasis. For the improvement of clinical efficacy and the decline in the incidence of treatment-related relapse, recent progress in developing novel techniques and effective disease management strategies for optimal targeting has led to the emergence of targeted alpha therapy (TAT) in GEP-NENs patients. For instance, labeled technology and combination therapy could contribute to significantly improved long-term outcomes. However, the exact dosimetry for precision oncology, the shortage of radionuclides, and the stability of disease control are still under careful consideration. More high-quality, large-scale prospective studies are essential for obtaining valuable evidence on challenging problems and for further exploration.

**Graphical abstract:**

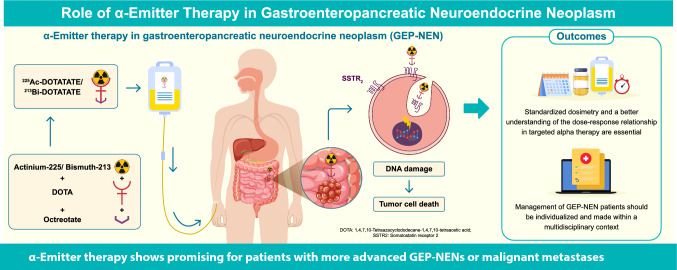

## Introduction

Gastroenteropancreatic neuroendocrine neoplasms (GEP-NENs) are the most frequent primary sites of neuroendocrine neoplasms (NENs),with an increasing worldwide prevalence and incidence over the last 3 decades, while the annual adjusted incidence was reportedly 3.53 cases per 100,000 inhabitants in Japan [1]. GEP-NENs are subdivided into two categories: NENs of the luminal gastrointestinal tract (mainly stomach 19.7% and rectum 12.7%) and pancreatic NEN (pNEN) (accounts for 33.5%) [2]. Up to 90% of GEP-NENs are characterized by overexpression and wide anatomic distribution of somatostatin receptors (SSTRs) on its cell membrane, mainly SSTR2, making it possible as an effective therapy target. Somatostatin analog (SSA) therapy such as octreotide or lanreotide is undoubtedly the first-line treatment option for midgut neuroendocrine tumors (NETs) based on the results of the octreotide (PROMID study) [3] and the CLARINET [4] studies (Lanreotide® anti-proliferative response in patients with GEP-NETs), demonstrating a significant prolongation of progression-free survival (PFS) and longer time to tumor progression compared to placebo [5]. Though SSA is effective in the symptomatic disease management and stabilization of well-differentiated disease, there were still some dilemma in the long-term surveillance, for instance, no predictive models and associated preventive measures are available in clinical situations, making it difficult to control the events of relapse and progression, especially in those GEP-NETs patients with poor-differentiated, advanced and rapid growth features. Furthermore, patients can become resistant to SSAs treatment without exact mechanisms when repeated treatments were needed [6]. Various therapeutic strategies on GEP-NENs and the associated SSTR focused around the use of radiopharmaceuticals with its good pharmacological characteristics and the favorable outcomes, especially peptide receptor radionuclide therapy (PRRT), have been developed rapidly.

## The β-particles- or α-particles-based PRRT for GEP-NENs patients

### Clinical use of PRRT with β-particles in patients with GEP-NENs

Surgery is not always possible in patients with GEP-NENs, because 50–60% of patients already present with metastatic disease when diagnosed [7]. In patients with locally advanced unresectable or metastatic NETs, treatment goals should focus on tumor growth control and symptom relief. PRRT was clearly stated as a treatment option for GEP-NENs by European Society of Medical Oncology in 2010, and American Society of Clinical Oncology guidelines recommend PRRT as a second-line treatment for metastatic intestinal NETs of G1/G2, if they are positive for SSTR expression [8, 9]. This therapy is particularly valuable when patients are resistant to chemotherapy or relapse to SSA, and can significantly extend both PFS and overall survival (OS) with a well-established safety profile. Currently, the most widely used β-particles isotope in PRRT is ^177^Lu, with a half-life of 6.7 days. PRRT can be conducted using specific targeting molecules labeled with either diagnostic radionuclides (γ-emitters) or three different types of therapeutic radionuclides (β^−^emitters: 497 keV at 78.6%, 384 keV at 9.1%, and 176 keV at 12.2%) [10]. NETTER-1 (2012–2016, *n* = 229) and NETTER-2 (2020–2022, n = 226), both were compelling evidence for multinational randomized phase III trials of PRRT, compared the clinical outcomes of ^177^Lu-DOTATATE treatment with high-dose octreotide long-acting release (LAR) in patients diagnosed as advanced GEP-NETs. Results showed that although ^177^Lu-DOTATATE treatment did not significantly improve median OS versus high-dose long-acting octreotide (48.0 months vs. 36.3 months, two-sided *p* = 0.30), but the PFS was prolonged significantly (65.2% vs 10.8%) in NETTER-1 [11]. This clinical research leaded to the approval of ^177^Lu for the treatment of SSTR positive GEP-NETs by the EMA (European Medicines Agency) in 2017 and FDA (Food and Drug Administration) in 2018 [12]. Furthermore, in NETTER-2, ^177^Lu-DOTATATE plus octreotide LAR significantly extended median PFS (by 14.3 months, *p* < 0.0001), suggesting that ^177^Lu- DOTATATE should be considered as the first-line therapy in patients with grade 2 or 3 advanced GEP-NETs [13].

Based on the promising data in Europe, PRRT has become widely known in NET patients in Japan. However, because of complex regulations for radiopharmaceuticals, it took nearly 10 years to obtain the approval for PRRT use by the Japanese government. Japanese NET patients who had indications for PRRT were transferred to Europe for treatment before ^111^Lu-DOTATATE (Lutathera) was covered by insurance in 2021 [14]. Complicated regulations for radiopharmaceuticals in Japan are still unresolved.

### Limitations of PRRT with β-particles

Although a meta-analysis by Kim et al. demonstrated that PRRT could achieve an average disease control rate of 82% with acceptable safety [15]. However, PRRT with β-particles also presents two main challenges which were found similar in the prior therapeutic strategies like chemotherapy or SSA: a lack of response to therapy and post-treatment relapse. The response rates are only 18–44% based on Response Evaluation Criteria in Solid Tumors (RECIST) criteria and 7–37% based on the Southwest Oncology Group (SWOG) criteria with rare complete remission when used alone [16, 17]. One of the reasons may be that hypoxic cancer tissues are resistant to β-emitters [18]. Only 26–55% of patients could achieve disease stabilization with ^177^Lu-DOTATATE therapy unfortunately, while 18–32% are refractory inevitable to the treatment. More data showed that patients who achieve disease stabilization invariably relapse within 2–3 years of starting PRRT [19]. Besides this, PRRT is not effective in patients if SSTR expression is negative, with low overall survival rates. These outcomes are related to their characteristics: a high proliferative index (Ki-67 > 20% or G3), less frequent expression of SSTR, and rare production of hormonal syndromes [8, 20]. Some studies also demonstrated some negative predictive factors for the efficacy of PRRT, including large lesions, high hepatic tumor burden, fluorodeoxyglucose avidity and high Ki-67 [21–23]. Some alternative options tried to provide for NETs patients with low or absent SSTR expression but positive expression for other types of receptors, for instance, cholecystokinin-2 (CCK2) receptors and glucose-dependent insulinotropic polypeptide (GIP) receptors [24, 25], would become new therapeutic targets for improvement of the clinical outcomes.

Another significant limitation of PRRT has a relationship with the inherent feature of β-particles. Due to their long path length, the minimal disease, including micrometastases or residual tumor tissue after surgical debulking did not exhibit a good response to β-particle therapy. Limited success has been reported in the treatment of minimal disease using low-LET radiotherapy [26, 27]. Furthermore, another limitation of quite long penetration range is the higher risk of damage to non-target tissues, mainly the kidneys and bone marrow, which have a significant impact on side effects [28]. Fortunately, nephrotoxicity is primarily associated with ^90^Y therapy but is rare with ^177^Lu therapy [29]. Transient hematotoxicity is more common in clinical practice, particularly thrombocytopenia, which typically occurs 4–6 weeks after the treatment cycle and resolve quickly. Long-term myelodysplasia or leukemia develops in 2% of patients [30].

## Clinical use of PRRT with α-particles in patients with GEP-NENs

### Efficacy of PRRT with α-particles in patients with GEP-NENs

α-Particles is characteristic with higher energy and shorter penetration range in comparison to β-particles. The clinical experience with SSTR-based targeted α therapy (TAT) in GEP-NENs patients showed very promising results even in those patients resistant or refractory to PRRT treatment with labeled β-particles [31]. Based on their physical properties, the optimal setting for α-particles therapy is probably the stage of micrometastases, early relapse, or the stage of minimal disease observed after surgical treatment or induction therapy in an attempt to eradicate residual tumor cells, serving as a valuable complement to β-particle therapy. To date, the commonly used α-emitters including ^225^Ac-DOTATATE, ^213^Bi-DOTATOC, and ^212^Pb-octreotate have been investigated for the treatment of GEP-NENs. Till now, there have been already six clinical researches for GEP-NENs treated with TAT (Table 1) [31–36]. The first human study of patients with progressive advanced neuroendocrine liver metastases refractory to ^90^Y/^177^Lu-DOTATOC treatment, which involved seven patients, demonstrated enduring responses when using therapeutically effective doses of ^213^Bi-DOTATOC. Recent early phase clinical studies have shown promising results for the treatment of metastatic NETs with ^225^Ac-DOTATATE and ^212^Pb-DOTAM-(Tyr3)-octreotate (DOTAMTATE), even in patients who do not respond to ^177^Lu-DOTATATE therapy.

**Table 1:** The summary of PRRT clinical trials using α-emitters for GEP-NENs patients with somatostatin-overexpressing

Authors/nationality/year	Indications(n)	Administration regimen Radiopharmaceutical; cumulative or mean activity; F = follow-up duration	Prior concomitant therapies (n)	Clinical responses	Adverse events
Ballal et al. [29] India.2023 A Real-World- Scenario	Well-differentiated, inoperable, or metastatic GEP-NETs (*n* = 91)	^225^Ac-DOTATATE (100–120 kBq/kg) Four cycles per patient F:24 months	Surgery(*n* = 20) Chemotherapy(*n* = 20) ^177^Lu-PRRT(*n* = 57)	mo OS probability: 70.8% 24-mo PFS probability: 67.5%	Grade 3 thrombocytopenia (*n* = 1)
Demirci et al. [30] USA.2023 Retrospective study	Grade 1/2 metastatic NETs (*n* = 11)	^225^Ac-DOTATATE (mean 8.2 ± 0.6 MBq)	^177^Lu-PRRT(*n* = 10) Chemotherapy(*n* = 11) SSA LAR(*n* = 10)	Disease control rate: *n* = 8 Stable response: *n* = 4 Partial response: *n* = 4 Progressive rate: *n* = 1 Median PFS: 12 months	Grade 2 renal toxicity and grade 2 hematotoxicity (*n* = 1)
Kratochwil et. al [31] Germany. 2021 Retrospective study	SSTR ( +) solid tumors(*n* = 39; GEP-NENs (*n* = 10))	^225^Ac-DOTATOC 20 MBq per cycle,4 monthly repetition Cumulative doses: up to 60–80 MBq	^177^Lu-PRRT(*n* = 32) Chemotherapy(*n* = 19) SSA LAR(*n* = 21)	Median OS: 20 months	Dose-dependent thrombocytopenia and leucopenia Kidney failure (*n* = 2)
Ballal et al. [32] India. 2020 Prospective study	Metastatic GEP-NETs (*n* = 32; Stable 44%; Progressive 56%)	^225^Ac-DOTATATE (100 kBq/kg) at an interval of 8 wks; F: 8 months	^177^Lu-PRRT	The morphological response: 75% (Partial remission: 62.5%; Stable disease: 37.5%)	Grade I/II hematologic toxicity (*n* = 20)
Kratochwil et al. [28] Germany. 2014 Retrospective study	Progressive GEP-NETs with liver metastases (*n* = 7)	^213^Bi-DOTATOC Cumulative doses: 15.8 GBq F: > 2 yrs	^90^Y/^177^Lu-PRRT	Enduring responses (*n* = 7)	Chronic kidney toxicity (*n* = 1) Hematotoxicity (*n* = 4) Graves’ disease (*n* = 1)
Delpassand et al. [33] USA.2022 Prospective study	Metastatic or inoperable NETs (*n* = 20)	^212^Pb-DOTAMTATE: incremental 30% dose increase, 4 cycles of 2.50 MBq/kg at 8-wks intervals (67.6 μCi/kg, *n* = 10)	SSA	Objective response (*n* = 8/10)	No serious treatment-emergent adverse events

*OS* Overall survival, *PFS* progression-free survival, *SSA* somatostatin analog, *LAR* long-acting release

### Strategy for improving the efficacy of TAT

The scope of radionuclide therapy has dramatically expanded with the development of high-affinity target molecules and novel chelating agents that can provide thermodynamically stable complexes in vivo [37]. An ideal radionuclide labeling method should be reliable, safe, and efficient with minimal impact on the original properties of the nanocarriers [38]. When selecting the final method, the compatibility between the radionuclide’s half-life and chemical activity, the characteristics of the nanomaterial, and the reaction conditions and time requirements of the labeling process must be considered.

**Fig. 1:**
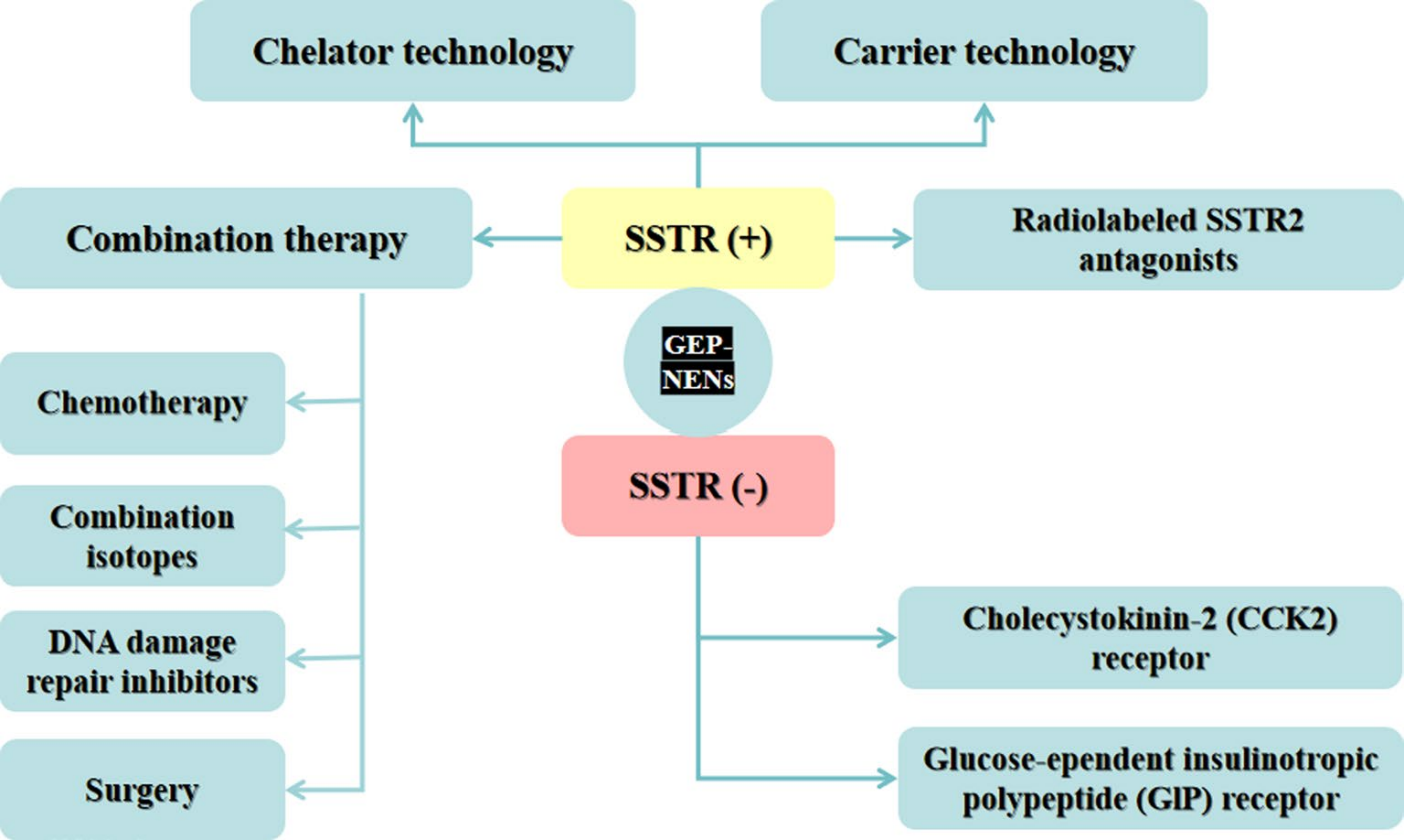
Refinements in improvement of PRRT with α-emitters

There are some strategies for improving the efficacy of TAT in the SSTR-expression GEP-NENs patients. When SSTR was detected positive, the evolutions of the properties of the radionuclide itself, the combination therapy with SSA or chemotherapy and the changed agonists for antagonists could be more effective in the clinical outcomes. When SSTR was detected negative, the PRRT with α-emitters based on the SSTR is no longer applicable for the GEP-NENs. New therapeutic targets underwent further researches (Fig. 1).

#### Developments in carrier technology

A major obstacle to using α-emitting radionuclides is their limited availability, which can be improved when using carrier technology. Optimizing the design and use of carrier technology can achieve targeted delivery of radionuclides, enhance tissue repair, and reduce drug toxicity. Combining α-emitting radionuclides with small drugs or biological macromolecules that target radiation-resistant proteins is particularly effective [39]. This approach is valuable because the sources of therapeutic resistance offer excellent targets for further improvement in the specificity of TAT. Common targeting molecules include monoclonal antibodies, small compounds, peptide and nanobodies [40].

Monoclonal antibodies are highly selective for antigens expressed in tumor cells, resulting in high tumor uptake and low accumulation in healthy tissues. However, monoclonal antibodies labeled with α-emitters often exhibit unsatisfactory pharmacokinetics owing to their large molecular size [28]. Despite the increasing number of drug candidates undergoing clinical trials, challenges persist in their development and adoption, including inadequate targeting, high non-target radionuclide uptake, and low target-to-non-target ratios [41]. Medical radionuclides are limited by their strict energy requirements, penetration, and half-lives, whose biocompatibility and in vivo stability require further investigation.

Small compounds and radiolabeled peptides offer several benefits, including accessible radiolabeling, straightforward chemical synthesis, rapid clearance from the circulation, rapid penetration, tissue distribution, and reduced immunogenicity [42]. Various agents serve as specific probes and vectors for α-emitting radionuclides, including substance P, anti-prostate-specific membrane antigen (PSMA) small molecule antibodies, and meta-aminobenzylguanidine(MABG) [43]. Owing to the short path length of α-particles and their minimal cell damage, small molecules with higher penetration may prove great advantages for tumor treatment. Moreover, they often demonstrate superior tumor penetration and faster clearance than monoclonal antibodies [44]. Peptide receptor-based TAT using labeled ^225^Ac and ^213^Bi combined with fast-diffusing, low-molecular-weight peptides, such as DOTATOC (DOTA-coupled Tyr3-octreotide), DOTATATE (1,4,7,10-tetraazacyclododecane-1,4,7,10-tetraacetic acid coupled Tyr3-octreotate), and substance P, as carriers is a promising strategy and is expected to play an increasingly important role in future clinical applications [27, 45].

However, the targeted effects would be diminished by the short stay in the tumor. It remains unclear whether small peptide-based targeted radionuclide therapy (TRT) can be broadly applied to other solid malignancies or if peptide modification strategies, such as bispecific binding motifs and stabilization techniques to improve binding affinity and in vivo stability, can further enhance TRT efficacy [46]. The success of TRT in solid tumors raises questions about whether this represents a paradigm shift in carrier molecule selection from monoclonal antibodies to small peptides and whether new treatment strategies or antibody engineering could improve monoclonal antibody-based TRT for solid tumors. For example, site-specific conjugation techniques for radionuclides and engineered antibody fragments for TRT require clinical testing [47]. PSMA-617 [48] and PSMA-I&T [49] are the two most extensively studied anti-PSMA ligands. PSMA-I&T, introduced in 2014, is a theranostic PSMA-targeting small molecule. *Mathias *et al*.* reported a promising anti-tumor effect of ^225^Ac-PSMA-I&T treatment in advanced metastatic castration-resistant prostate cancer (mCRPC), with reduced grade 3/4 hematological side effects, suggesting that this may be an additional therapeutic option for patients with end-stage mCRPC [50].

Compared to conventional monoclonal antibodies, nanobodies offer several advantages: the ability of effective tumor penetration with remarkable stability; small size which allows them to easily cross the blood–brain barrier and facilitate the identification and binding of hidden tiny radionuclides; high affinity and specificity with simple structure; low immunogenicity and rapid clearance from the blood and kidneys [51]. These properties make nanobodies promising candidates for TAT. The retention and confinement of α-emitting radionuclides do not always depend on the size of the nanoparticle system. It is important that the nanoparticulate-embedded α-emitters exhibit sizes within the 50–150 nm range to be able to penetrate the tumor vasculature more effectively. Inorganic core nanoparticles loaded with α-emitters and surrounded by confinement layers seem to be the most efficient alternative, although further research is needed to determine the appropriate thickness of the confinement layers [52].

#### Chelators

Novel chelators play important role in the efficacy of TAT, which can provide the α-emitters thermodynamically stable complexes in vivo [53]. However, the effects of α-emitters have been limited due to the relative lack of optimal chelators. Furthermore, not all radionuclides were suitable for chelation, such as ^223^Ra and ^211^At decay products [54, 55]. To date, two types of improved chelator stability are commonly used in clinical applications, including bifunctional chelating agent (BFCAs) and macrocyclic ligand. BFCAs, such as DOTA, DOTATATE, and DOTATOC, can build a stable link radiometal to carrier molecules as a component of radiopharmaceuticals. They can exhibit high thermodynamic and excellent in vivo stabilities [56], which are currently used to treat NETs with TAT. Besides, the macrocyclic ligand macropa exhibits superior labeling characteristics for ^225^Ac radiometals compared to other ligands [57]. Similarly, macropa shows promising as an effective chelator with ^223^Ra^2+^ therapeutic radiometal [58]. Recent advance on the new chelator with ^212^Bi coupling can also improve the therapeutic index for targeting ligands (known as Pb-Specific-Chelator or PSC) [59, 60]. The effective ^211^At-labeling using arylboronic acids/esters and the exploration of boron cage reagents could improve the stability and distribution [55]. Issues relating to suitable chelating agents and chelation chemistry to sequester this element in vivo remain to be solved, as none of the radiolabeling methods investigated, thus, far have been proven to efficiently form the required complex.

H4noneunpaX, a new chelating ligand with an unusual diametrically opposed arrangement of pendant donor groups, has been developed to this end, which shows significant potential for theranostic applications involving ^225^Ac/^155^ Tb or ^177^Lu/^155^ Tb [61].

#### Combination therapy

Clinical applications of TAT are expanding with various radionuclides and combination therapies, including chemotherapy, DNA damage-repair inhibitors, combination isotopes, and surgery in diverse clinical settings [62–64]. Before the introduction of molecular targeted agents, systemic chemotherapy was recommended for G1 or G2 NENs with a high tumor load or those displaying significant tumor progression in less than 6–12 months, as well as for G3 NENs, in clinical practice. However, response rates of at most 30–40% in patients with small bowel NENs highlighted the limited effects of these treatments.

With the development of molecular targeted therapies, the combination of TRT with mammalian target of rapamycin (mTOR) shows promising advantages for future treatments [65]. The mTOR pathway is involved in NETs growth and DNA damage, and has proven to be an effective target in patients with advanced GEP-NENs. Everolimus has been approved by the FDA and the European Medicines Agency for the treatment of advanced renal cell carcinoma (RCC), subependymal giant cell astrocytoma (SEGA) associated with tuberous sclerosis (TSC), pancreatic neuroendocrine tumors (PNET) [66]. The results from the RADIANT trial demonstrated that patients with advanced G1 or G2 pancreatic and intestinal NENs had significantly prolonged PFS with everolimus relative to that with placebo, implying its anti-proliferative action in different NENs [67, 68]. Other DNA damage-repair inhibitor including poly ADP-ribose polymerase (PARP) inhibitor, ribonucleotide reductase (RNR) inhibitor, were also found to enhance anti-tumor effects to some extent when combination with TAT [69].

Surgical intervention in advanced stages of NENs has shown benefits for some patients [70]. However, studies reveal a significant risk of recurrence after radical surgery. A single-center study of 615 patients with small intestinal neuroendocrine tumors (SiNET) demonstrated this risk [71]. Another study of 441 patients (224 with PNET and 217 with Si NET) found that about 30% of patients with NETs experienced recurrence within 5 years of radical surgery [72]. Combination with TAT maybe an effective way to control the incidence of recurrence. In addition, ^177^Lu-DOTATATE was reported to convert 15 out of 57 (26.3%) unresectable primary GEP-NETs into resectable ones in a small non-controlled study [73].

Besides the above, the combination of both isotopes may, therefore, be considered for patients with tumors of various sizes and non-homogeneous receptor distribution, especially ^177^Lu and ^90^Y [74].

#### Radiolabeled SSTR2 antagonists

Antagonist peptides are increasingly being developed with possible superior biological properties as compared to the agonists, making them suitable candidates for PRRT. Baum et al. [75]. found that ^177^Lu-DOTA-LM3 (SSTR antagonist) had a higher uptake and longer effective half-life than ^177^Lu-DOTATOC (an SSTR agonist). This antagonist proved more effective in treating advanced metastatic NENs, particularly in patients with low or no SSTR agonist binding. All patients tolerated the therapy without any serious acute adverse effects [75–78].

#### Exploration of new targets

For those GEP-NENs patients with low or absent SSTR expression but positive expression for other types of receptors, such as CCK2 receptors [24], GIPR [79], attracting attention as new targets for effective therapy. Recent data in mice model demonstrated a novel rationally designed PET radioligand, [^68^Ga]Ga-C803-GIP, showed binding characteristics and specificity towards the GIPR, implying promising application in future [80].

## Current limitations of TAT

### Dosimetry

The optimization in any targeted therapy would be to maximize anti-tumor efficacy while keeping the risk of toxicity below an acceptable level. Given the high relative biological effectiveness (RBE) of the α-particle, many TATs are characterized by an inhomogeneous distribution of radiopharmaceuticals. The biodistribution is of heightened importance to ensure targeted delivery and the minimum off-target exposure. The dosimetry is very crucial to the maximum safe cumulative activity administered for maximal efficacy. However, the accurate dosimetric data is a major challenge in internal radiotherapy. Calculating the administered activity of a treatment fraction based on body weight can result in overtreatment (causing high toxicity) or undertreatment (producing no clinical effects) in many patients [81]. Currently, conventional approaches are often limited to average parameters, and there are no dosimetric tools available to accurately estimate the target and the non-target organ-absorbed tumor doses and maximum tolerable dose related to TAT. A phase I clinical trial in which patients received up to 925 kBq of ^213^Bi reported α-particle doses of 1 Gy in the blood. However, microdosimetric calculations revealed that the actual dose to important cell targets was much lower (2 cGy for endothelial cells and 10 cGy for lymphocytes), less than 10% of the macroscopic dose to the blood [82]. γ-Photons could be emitted during many α-emitting radionuclides’ decay, which are valuable for post-therapy imaging and dosimetry [83]. These functions provided by the same radionuclide are considered as the main-stream of theragnostic.

Meaningful dosimetry studies with TAT require detailed information on the target geometry, as well as pharmacokinetic data of the α-emitting radionuclide and the possible fate of daughter particles at cellular and subcellular scales. This has become an active area of research with several advances in clinical imaging and dosimetry in recent years [84]. *Hindorf *et al. defined the imaging characteristics and feasibility of ^223^Ra, which enabled the study of its biodistribution and pharmacokinetics in patients [85]. In addition, *Abou *et al. confirmed in animal models that ^223^Ra is deposited on the bone surface surrounding tumors and that skeletal accumulation is dependent on local blood vessel density [86, 87]. Besides the therapeutic function by β-emitters, γ-emitters could be used for diagnosis, which can provide accurate pharmacokinetics of a radiopharmaceutical administered for dosimetric estimation using the same radiometals.

### Side effects

Generally, PRRT is considered to be a safe treatment option. For most studies involving long-lived α-emitters, recoiling daughters pose a serious problem and toxic effects are likely, though the balance between anti-tumor effect and toxicity will vary and must be understood for each isotope, targeting ligand, and indication [88]. The α-particle emissions from radionuclides create a recoil effect. The energy of this effect is 1, 000 times greater than that of the chemical bond [88], which causes the radionuclide to break free from its original molecule. Consequently, daughter products can travel throughout the body, accumulating in off-target organs such as the kidneys and bones, which remain an ongoing challenge [89]. Notably, when treated with [^225^Ac]Ac-DOTATATE, low-grade hematological toxicity was reported as the most common treatment-related side effect in a small subset of patients with anemia (14.39%), leukocytopenia (4.12%), and thrombocytopenia (7.18%) [28]. Other troublesome side effects of TAT are hyposalivation and xerostomia, which are mainly caused by the uptake of ^225^Ac-PSMA-617 by the salivary glands [90]. New approaches are needed to reduce these toxic side effects. Some studies demonstrated that the dynamic de-escalation and cocktail approaches may improve tolerability without losing excessive anti-tumor activity [83].

### Shortage of radionuclides

The production methods range from nuclear reactors to cyclotrons and generator systems. More efficient production of α-emitting radionuclides should be explored to increase their availability. Therefore, developing new supply chains is critical. For instance, ^225^Ac is not available in sufficient quantities for commercial use because of the lack of large-scale ^233^U production [82]. Longer half-lives reduce waste generated during radiochemical processing and distribution. However, it would be challenging for the preparation of the ^213^Bi due to the quite short half-lives. Generator-produced radionuclides offer a convenient way to create short-lived isotopes on-site through the decay of long-lived parents when available. The high-energy γ emission of 208Tl (Eγ = 2.6 MeV) necessitates heavy shielding to reduce radiation exposure, limiting its clinical use [81]. In contrast, ^225^Ac and ^213^Bi can be reliably produced from established generator systems with a high specific activity and purity. Their availability in clinical settings, independent of local reactors or cyclotron facilities, and favorable chemical properties allow for the synthesis of stable radio-conjugates using established chelate molecules. A large amount of radioactive waste is produced along the way, and transportation is a public safety issue when nuclides are obtained via nuclear reactors. The limited number of cyclotrons poses logistical problems for the delivery of short-lived radionuclides. The use of linear accelerators does not always make it possible to obtain radionuclides of sufficient purity and activity [89].

As mentioned above, PRRT with α-emitter could exhibit effective roles in those advanced GEP-NENs patients who are resistant or refractory to treatment with β-emitters. However, low incidence and high variability hamper the implementation of high evidence trials. There are several kinds of strategies to improve the efficacy of TAT, including modification of α-emitter’s properties with different chelators or carriers, combination with other therapies and exploration of new targets, which demonstrate a promising application in GEP-NENs patients. However, there should not be a “one-size-fits-all” approach for TAT. The chosen radionuclide should match the requirements of the indication being treated. Questions persist regarding the optimal injected activity, potential benefits of activity fractionation and dose repetition and ideal therapeutic sequence. Careful consideration is needed for standardized dosimetry and a deeper understanding of the dose–response relationship in TAT. Studies explore various biomarkers, genetic and epigenetic alterations as prognostic factors, and treatment response predictors in NENs as an alternative choice for dosimetry, including circulating tumor cells (CTCs), circulating tumor DNA (ctDNA), histone modifications and miRNAs, as prognostic factors and predictors of response to treatment [91]. A recently developed multigene liquid biopsy (NETest), a novel multigene liquid biopsy, shows promising in assessing NEN surgical removal, predicting aggressive behavior, and evaluating SSA and PRRT efficacy [92]. Novel therapies are being investigated in multiple ongoing clinical trials. Meanwhile, current efforts focus on personalized treatment and precision oncology, targeting specific genetic and protein regulators of neoplasms. The management of patients with NENs should be individualized, with decisions made in a multidisciplinary context——all to improve patient’s outcomes.
